# Effects of different exercises on health-related physical fitness among middle-aged and elderly women

**DOI:** 10.3389/fpsyg.2025.1631702

**Published:** 2025-08-04

**Authors:** Xiaorong Bai, Yuhui Wang, Soh Kim Geok, Zongqiang Jin, Wensheng Xiao, Junlong Zhang

**Affiliations:** ^1^College of Sports Training, Tianjin University of Sport, Tianjin, China; ^2^School of Physical Education, Huzhou University, Huzhou, China; ^3^Department of Sports Studies, Faculty of Educational Studies, Universiti Putra Malaysia, Serdang, Malaysia; ^4^College of Physical Education, Hunan Normal University, Changsha, China

**Keywords:** Tai Chi Chuan, walking, Jiamusi, middle-aged and elderly women, physical fitness

## Abstract

**Objective:**

To explore the effects of long-term exercise of Tai Chi Chuan, walking, and Jiamusi gymnastics on the health-related factors of physical fitness among middle-aged and elderly women.

**Methods:**

Long-term exercise Tai Chi Chuan group, walking group, Jiamusi gymnastics group, and non-exercise group were used to measure their health-related physical fitness, balance, and life satisfaction through long-term exercise Tai Chi Chuan group, walking group, Jiamusi gymnastics group, and non-exercise group matched by age, height, and weight.

**Results:**

Compared with control group, Tai Chi Chuan were significant differences in all indicators, except systolic blood pressure (SBP), diastolic blood pressure (DBP), resting heart rate (RHR), and life satisfaction (*p* < 0.05). Walking were significant differences in RHR, force vital capacity (FVC), waist–hip ratio (WHR), sit and reach (SR), handgrip strength (HGS), and chair stand test (CS; *p* < 0.05). Jiamusi gymnastics had a significant difference in FVC, hip circumferences (HC), SR, HGS, and back scratch (BS). Compared with different exercise group, Tai Chi Chuan exercises significantly improved balance, SR, and WHR. Jiamusi gymnastics had a statistically significant impact on RHR. The CS was statistically impacted by walking.

**Conclusion:**

Middle-aged and older women are affected differently by different types of exercise. When the three groups—Tai Chi Chuan, walking, and Jiamusi gymnastics—were compared, it was discovered that Tai Chi Chuan had a greater impact on improving balance and trunk flexibility, Jiamusi gymnastics had a greater impact on improving cardiopulmonary fitness, and walking was more effective at strengthening the lower limbs.

## Introduction

At the moment, every country in the globe is confronting a variety of difficulties and challenges caused by an aging population. Due to China’s large population base, according to data from the National Bureau of Statistics of China, the population of China aged 65 or older will reach 194 million by the end of 2022, accounting for 25% of the world’s elderly population (Yifu et al., 2024). Thus, China’s strategy to deal with aging has positive reference significance for all countries.

In China, over 78% of people over 60 currently have chronic illnesses, and these illnesses are getting worse at younger ages. With rising average life expectancy and an older population, preventing and treating chronic diseases has become a critical aim of Healthy China (Chen et al., 2022). Chinese government is actively promoting ways for the elderly to exercise, increasing sports function for preventing disease and maintaining health (Guo et al., 2016). Physical exercise is a healthy and affordable form of exercise. It can not only promote health and prolong life, but also play an important role in the prevention and treatment of many diseases (Dhuli et al., 2022). The World Health Organization (WHO) recommends about 150 min of moderate-intensity physical activity or exercise per week, this could reduce the risk of all-cause death, cardiovascular disease death, high blood pressure, site-specific cancers, type 2 diabetes and fall prevention, mental health promotion (reducing anxiety and depression symptoms), cognitive health and obesity measures may also be improved (WHO, 2022). At the same time, research has confirmed that physical exercise can improve the health and quality of life of the elderly by improving their physical and mental state. Thus, the elderly frequently choose physical exercise based on their hobbies, but different types of exercise have different effects on their health. Tai Chi Chuan, walking, and square dancing are the current trends in middle-aged and older populations’ exercise habits. Tai Chi Chuan, as one of the core categories of Chinese martial arts, was originally established for combat and self-defense. It integrates the philosophy of yin-yang with the theory of meridians in traditional Chinese medicine, forming a practical system that uses softness to overcome hardness and cultivates both internal and external aspects. It has now become a sport and exercise method for most practitioners. Over 500 million people practice Tai Chi Chuan in about 150 nations and regions worldwide as of September 2024, and its reach is still growing. Tai Chi Chuan can improve the elderly’s balance function, running can enhance the elderly’s cardiopulmonary function (Tan et al., 2022), participation in square dancing can effectively improve middle-aged and older adults’ self-efficacy, social support, and positive psychological qualities (Liu et al., 2024; Ou et al., 2022). Currently, Jiamusi gymnastics of square dance is an aerobic fitness workout that combines radio calisthenics, fast walking, dancing, and traditional health preservation. Its simplicity, quick tempo, clear impact, and appropriateness for all age groups have led to its quick national and international popularity. It has opened branches abroad in Australia and the United States. It is currently one of the exercise regimens that the Chinese General Administration of Sport promotes. As of 2023, Jiamusi gymnastics of square dance had accumulated an average of over 100,000 daily followers on platforms such as Douyin. Walking improves mental health, sleep quality, and longevity while lowering the risk or severity of a number of health outcomes, including dementia, type 2 diabetes mellitus, cardiovascular and cerebrovascular disorders, and cognitive impairment (Ungvari et al., 2023). Today’s women in particular are expected to fulfill a variety of responsibilities, including those of wives, mothers, caretakers, and devoted workers; as these expectations increase, PA levels decline even further. To assist middle-aged (aged 50–60 years) and older adults (aged 60 years and older) women in selecting suitable exercise regimens to help reduce the incidence of various chronic diseases, it is necessary to compare the most common forms of exercise (Tai Chi Chuan, walking, Jiamusi gymnastics) among these populations.

Indicators such as satisfaction with life scale (SWLS) (Diener et al., 1985), balance, and health-related physical fitness (HRPF) were the focus of this study since they are strongly correlated with older individuals’ physical and mental health. Healthy levels of HRPF allow individuals to perform physical activities with vigor and promote resistance to fatigue (Cantell et al., 2008). Among the most extensively used tools for measuring overall life satisfaction, the SWLS is also one of the most well-liked surveys in the field of well-being and quality of life (Stover, 2022; Jovanović et al., 2020). Impaired balance are considered risk factors of incident falls (Rinkel et al., 2019). Thus, this study compares the HRPF, balance and life satisfaction of the middle age and elderly people who attended various sports items (Tai Chi Chuan, walking, Jiamusi gymnastics) for an extended period to determine which workout items have a greater impact on which physical functions, to propose effective and appropriate exercise items for the elderly with chronic diseases.

## Materials and methods

Writing and gather research data in accordance with the STROBE cross-sectional standards.

### Study population

In this cross-sectional study. Cluster sampling was used to recruit the elderly from all activity programs, whereas the control group was chosen by the community. The inclusion criteria for older adults in all groups were as follows: (1) Women aged 50–70 years; (2) Engagement in regular exercise for at least 6 months, no less than 180 min/week and moderate intensity, and no less than 3 times/week; (3) No evident disorders, such as neurological, cardiovascular, mental, or metabolic, before the exercise. The following were the criteria for exclusion: (1) Participants who took more than three drugs per day or who had physical or cognitive conditions that would limit their ability to exercise were excluded from the study; (2) Older adults participate in more one forms of physical activity; and (3) Those who had undergone heart or four-limb surgery within the previous 6 months are not eligible. Dropout criteria were the elderly who had not completed all the questionnaires and test items. Recruitment from July 10 to July 18, 2021, 104 volunteers aged 50–70 years old were recruited and categorized into Tai Chi Chuan group (*n* = 26), walking group (*n* = 26), Jiamusi gymnastics group (*n* = 26), and control group (*n* = 26).

All eligible literate participants supplied written informed consent, while the researcher read the consent statement to the illiterate individuals and signed it after getting their agreement. The study protocol was approved by Universiti Putra Malaysia’s Ethics Committee (JKEUPM-2020-296).

### Exercise

Tai Chi Chuan, walking, and Jiamusi gymnastics practices take the form of self-organized clubs. The control group did not exercise, but they continued with their daily schedule as usual.

The specific types of exercise style are as follows: Activities centered around Tai Chi Chuan include Yang-style Tai Chi Chuan and Chen-style Tai Chi Chuan. Jiamusi gymnastics include shoulders, upper limbs, chest, sides, belly, waist, body rotation, lower limbs, and body shaping. Walking carried out in open squares and parks accompanied by music.

Frequency of all exercise: Every day (unless there is severe weather).

Each club has a main organizer who oversees its leadership. Music is being played while exercising. Tai Chi Chuan and Jiamusi gymnastics are done in the morning (6:00–7:00 a.m.) and walking in the morning (5:30–6:30 a.m.), at a park or plaza.

### Outcome evaluation

Data collection dates are from July 19 to July 22, researchers measured HRPF and life satisfaction. Participants were instructed to refrain from excessive physical activity and acquire enough rest 24 h before the evaluation.

The test evaluated circulatory health, body composition, flexibility, muscular fitness, balance, and life satisfaction. The components of circulatory health include force vital capacity (FVC), resting heart rate (RHR), and blood pressure. Waist–hip ratio (WHR), hip (HC), and waist circumference (WC) are measurements of body composition. Comfort zones include sit-and-reach (SR) and back starch (BS). Handgrip strength (HGS), and 30-s chair stand test (CS) are examples of muscular fitness. Standing on one leg with closed eyes allows one to analyze balance. The Satisfaction with Life Scale is used to measure life satisfaction.

The specific operational measures were measured by citing the “Health-related physical fitness assessment manual” (ACSM, 2010), “Chinese National Physical Fitness Test Standard Manual” (Department of Mass Sports of the State Sports General Administration, 2003), and “Senior Fitness Test Manual Second Edition” (Rikli and Jones, 2013). The testing process and standards are carried out in accordance with the standards of ACSM testing (ACSM, American College of Sports Medicine, 2014).

During testing days, all equipment was calibrated on-site, and testers were capable of doing these measurements. Because there is no official order for measures, participants’ resting state-related components were evaluated first, followed by subsequent measurements in a suitable order. The Chinese-language satisfaction with life measure assessed well-being. This scale based on Satisfaction with Life Scale of Diener (Diener et al., 1985). The scale consists of five statements, each rated on a seven-point Likert scale. Total scores ranged from 5 to 35, with higher levels indicating increased life satisfaction.

### Statistics

Descriptive statistics were used to present the demographic characteristics of the total population in the four groups. Data cleaning and hypothesis testing were performed prior to analysis. Researchers use descriptive techniques for quality checking, including identifying missing data, outliers, and coding errors. Shapiro–Wilk and Levene’s tests were used for normal distribution and homogeneity test of variance for the continuous data, respectively. Continuous variables are presented as mean ± SD. One way ANOVA was used to evaluate the differences in HRPF, balance and life satisfaction data among the four groups, and the Bonferroni *post-hoc* test was used to determine significant differences between groups if there are significant differences overall. Results were considered statistically significant if their two-tailed *p* < 0.05. IBM SPSS Statistics software for Mac (version 27; IBM Corporation, Somers, NY, United States) was used for statistical analysis.

### Results

The multiple samples mean comparison formulas were utilized to determine the study’s sample size. Tai Chi Chuan, square dancing, the control group, and life satisfaction indicators mean (standard deviation) are 12.05 (1.96), 13.07 (1.51), and 9.04 (1.54), under earlier research that is comparable to this study (Su and Zhao, 2021). Known α=0.05 , β=0.1 . Utilizing the formula for determining an effective sample size.


$$n=\psi^{2}(\sum S_{i}^{2}/g)/[\sum {\left(\bar{X_{i}}/\bar{X}\right)}^{2}/(g-1)]$$

$$\bar{X}=(12.05+13.07+9.04)/3=11.39$$

$$\sum S_{i}^{2}=1.96^{2}+1.51^{2}+1.54^{2}=8.49$$

$$\sum {\left(\bar{X_{i}}/\bar{X}\right)}^{2}={\left(\begin{array}{l} 12.05-\\ 11.39 \end{array}\right)}^{2}+{\left(\begin{array}{l} 13.07-\\ 11.39 \end{array}\right)}^{2}+{\left(\begin{array}{l} 9.04-\\ 11.39 \end{array}\right)}^{2}=8.78$$

Known,α=0.05,β=0.1,ν1=3−1=2,ν2=∞,check tableψ=2.52

$$n_{\left(1\right)}=2.52^{2*}(8.49/3)/[8.78/(3-1)]=4$$

ν2=2(4−1)=6,check tableψ=5.14

$$n_{\left(2\right)}=5.14^{2*}(8.49/3)/[8.78/(3-1)]=17.03$$

Thus, each group 18 volunteers at least, consider drop-out and uncomplete test rate, in our research 26 volunteers for each group, total four groups 104 participants.

### Demographic characteristics

The Tai Chi Chuan, walking, Jiamusi gymnastics, and control groups included 104 participants with average ages of 57.00 ± 5.61, 56.77 ± 6.78, 59.31 ± 6.47, and 57.85 ± 6.21, respectively. There are over 60 (*n* = 40) and under 60 (*n* = 64). Except for occasional walking, the majority of Tai Chi Chuan, walking, Jiamusi gymnastics, and control group participants reported no other regular exercise. The results (Table 1) revealed that four groups were not significantly different in terms of age (*F* = 0.869, *p* = 0.46), height (*F* = 1.021, *p* = 0.387), weight (*F* = 1.584, *p* = 0.198).

**Table 1:** Demographics of the participants in the four groups.

Variables	Groups				F	*p*
	Tai Chi Chuan (*n* = 26)	Walking (*n* = 26)	Jiamusi (*n* = 26)	Control (*n* = 26)		
Age (year)	57.00 ± 5.61	56.77 ± 6.78	59.31 ± 6.47	57.85 ± 6.21	0.869	0.46
Height (cm)	159.49 ± 6.02	160.97 ± 6.03	158.96 ± 2.28	158.78 ± 4.74	1.021	0.387
Weight (kg)	62.44 ± 11.88	65.97 ± 6.72	66.66 ± 5.75	63.98 ± 4.86	1.584	0.198

Compared with control group, Tai Chi Chuan had better impact on all the measures, except for SBP, DBP, RHR, and life satisfaction (*p* < 0.05); walking had better effects on FVC, HGS, and CS (*p* < 0.05); Jiamusi gymnastics had a significant effect on FVC, HC, SR, BS, HGS (*p* < 0.05). Moreover, the effects of Tai Chi Chuan were significantly superior to walking on SR and balance; Tai Chi Chuan considerably outperformed Jiamusi gymnastics in terms of WHR and balance; walking has considerably better impacts on WHR and CS than Jiamusi gymnastics, and Jiamusi gymnastics were substantially superior to walking on RHR and SR (*p* < 0.05; Table 2).

**Table 2:** Results of health-related physical fitness and life satisfaction.

Test battery	Groups					
	Tai Chi Chuan (*n* = 26)	Walking (*n* = 26)	Jiamusi (*n* = 26)	Control (*n* = 26)	F	*p*
Circulatory health						
SBP (mmHg)	128.27 ± 9.323	121.42 ± 12.031	127.00 ± 6.847	127.92 ± 9.948	2.819	0.043
DBP (mmHg)	77.54 ± 8.358	76.38 ± 3.879	73.38 ± 6.450	74.23 ± 6.029	2.335	0.078
RHR (bpm)	71.96 ± 7.743	75.00 ± 5.075	66.96 ± 13.298	72.19 ± 8.109	3.541	0.017
FVC (mL)	2907.31 ± 368.999^⋆^	3112.69 ± 685.302^⋆^	2998.15 ± 264.842^⋆^	2129.81 ± 454.537	27.699	0.001
Body composition						
WC (cm)	82.012 ± 10.208^⋆^	86.792 ± 6.536	91.150 ± 10.030	92.800 ± 5.659	8.698	0.001
HC (cm)	97.562 ± 9.278^⋆^	100.746 ± 6.376	97.119 ± 5.465^⋆^	102.973 ± 2.914	4.830	0.004
WHR	0.839 ± 0.045^Δ,⋆^	0.862 ± 0.0445^*^	0.943 ± 0.1369	0.901 ± 0.0455	8.765	0.001
Flexibility						
SR (cm)	17.569 ± 7.344^#,⋆^	6.631 ± 8.956^*^	12.412 ± 5.103^⋆^	5.431 ± 8.136	13.122	0.001
BS (cm)	1.777 ± 6.376^⋆^	−0.988 ± 7.284	0.027 ± 5.415^⋆^	−5.735 ± 9.826	4.357	0.006
Muscular fitness						
HGS (kg)	28.561 ± 3.237^⋆^	26.238 ± 4.538^⋆^	27.565 ± 1.569^⋆^	23.004 ± 5.238	9.927	0.001
CS (rep)	20.54 ± 5.68^⋆^	23.00 ± 3.323^*,⋆^	18.96 ± 3.026	16.31 ± 3.496	12.674	0.001
Balance (s)	14.133 ± 8.679^Δ,#,⋆^	5.204 ± 2.253	6.378 ± 1.0496	3.865 ± 2.9491	22.532	0.001
Life satisfaction	30.50 ± 3.797	29.50 ± 3.808	30.04 ± 3.181	27.81 ± 3.889	2.655	0.053

DBP, diastolic blood pressure; SBP, systolic blood pressure; RHR, resting heart rate; VC, vital capacity; WHR, waist-to-hip ratio; SR, sit and reach; BS, back scratch, HGS, handgrip strength; CS, chair stand; ^Δ^Tai Chi Chuan vs. Jiamusi, ^#^Tai Chi Chuan vs. walking, ^*^walking vs. Jiamusi, ^⋆^all exercise groups vs. control.

## Discussion

**Figure 1:**
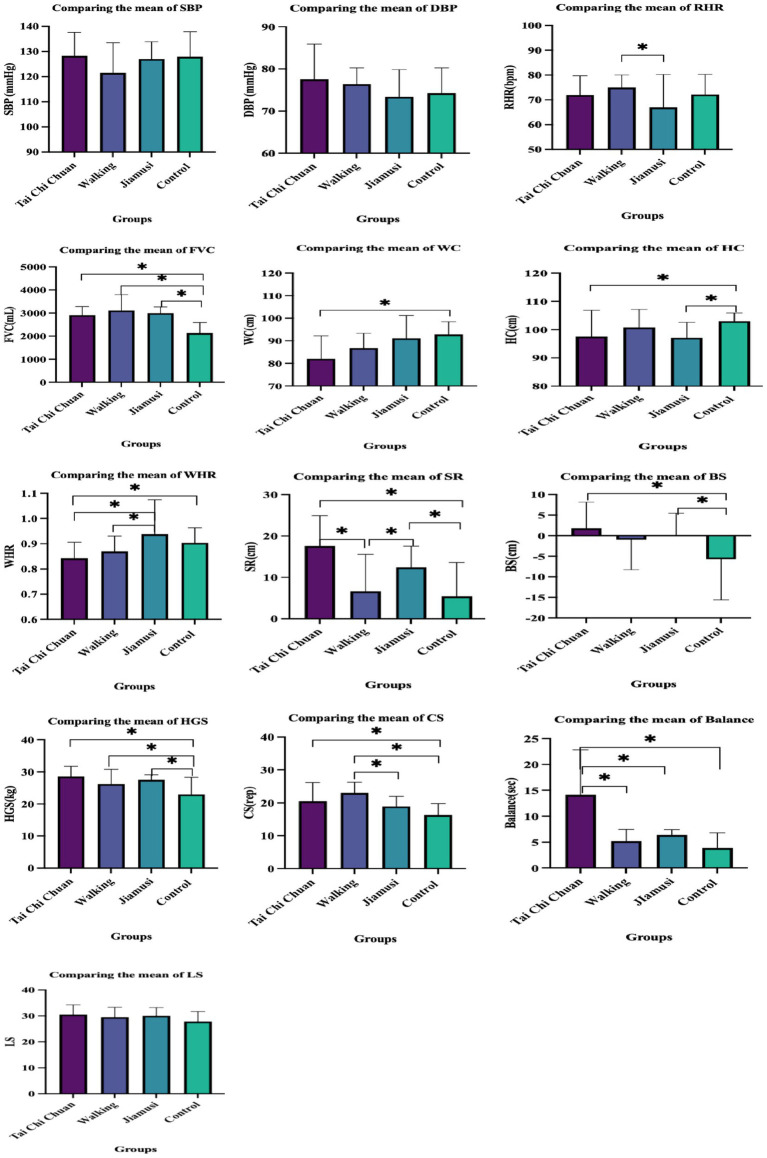
Comparing the mean of different groups.

This study aimed to assess the effects of long-term Tai Chi Chuan, walking, and Jiamusi gymnastics on health-related fitness, balance, and life satisfaction in middle-aged and elderly women. Research had showed that Tai Chi Chuan, walking, Jiamusi gymnastics had a significant effect on health-related physical fitness (HRPF), balance, and life satisfaction compared with control group. The benefits of each workout pattern vary by activity group, the details have shown in Figure 1.

The first is the comparison between the exercise group and the non-exercise group. When comparing the Tai Chi Chuan group to the non-exercise group, it was revealed that Tai Chi Chuan had positive impacts on forced vital capacity (FVC) of Circulatory health, body composition, flexibility, muscular fitness, and balance. However, Tai Chi Chuan was found to have no influence on systolic blood pressure (SBP), diastolic blood pressure (DBP), and resting heart rate (RHR) when compared to the control group. However, the exercise group’s SBP, DBP, and RHR were all within a healthy range. Moreover, Tai Chi Chuan movements involve all body parts and must be closely combined with breathing. Taking into attention the balance and stability of the body posture, coherence, and softness, through repeated practice, can enhance muscle strength, balance, and coordination ability (Xinyue, 2024). This is similar with earlier research findings, which have shown that Tai Chi Chuan improves cardiopulmonary fitness (Zheng et al., 2015), muscle strength and endurance (Bai et al., 2023), flexibility (Yasol-vicente, 2021), and body composition (Larkey et al., 2018). As a result, Tai Chi Chuan can be suggestion as a way in middle-aged and elderly women to prevent diseases and increase physical fitness.

Walking, compared to the control group, improves FVC and muscle fitness. Walking can boost oxygen intake, facilitate gas exchange in the lungs, and improve lung function (Roman et al., 2016). Walking causes the body to expend more energy, which helps to eliminate body fat and enhance muscular mass. Walking using certain actions, such as heel-first landing and toe spring, helps strengthen the lower limb muscles and ligaments (Wang et al., 2024). However, walking cannot improve significantly on SBP, DBP, RHR, body composition, flexibility, balance, and life satisfaction. SBP, DBP, and RHR readings are within a healthy range. Exercise by itself might not change body composition, but research indicates that it can be effective when combined with a healthy diet (Eglseer et al., 2023). The poll discovered that persons who walk frequently rarely stretch before and after exercise, which could be the main cause for their inability to improve flexibility (Bai et al., 2022). Walking did not significantly increase balance or life satisfaction, although it did enhance them when compared to the control group’s mean. According to studies, higher-intensity exercise is associated with life satisfaction, thus moderate and low-intensity walking has a minor impact (An et al., 2020). As a result, walking can be employed as a kind of exercise for the elderly to enhance their physical health, particularly in terms of muscle fitness and cardiovascular function.

Jiamusi gymnastics group outperformed the non-exercise group in terms of FVC, hip circumference (HC), flexibility, and handgrip strength (HGS). However, no significant benefit was observed in terms of waist circumference (WC), lower limb muscle strength, balance, and life satisfaction. Jiamusi gymnastics fitness exercise combines radio gymnastics, walking, dancing, and traditional health, as well as music, to form a comprehensive set of marching aerobics lasting 30 min and moving to people’s shoulders, necks, waists, knees, ankles, and other joints (Shengkui and Ping, 2016). Jiamusi gymnastics activity is a type of physical activity with undeniable benefits for middle-aged and older individuals. Although it has no effect on body composition or lower limb muscular strength, it is also caused by the movement features of the Jiamusi gymnastics. The moderate intensity of Jiamusi gymnastics workouts should be increased if weight loss is your goal.

To investigate the impact of various exercise methods on the fitness of middle-aged and older individuals, the three methods were compared, and it was discovered that each method placed a distinct emphasis on the influence of physical fitness. According to the Figure 1 and Table 2, distinct types of exercise have distinct movement characteristics, and their impact on human health also varies (Pesce, 2012).

Compared with other exercise groups, long-term Tai Chi Chuan exercise had a significant effect on waist–hip ratio (WHR), sit and reach (SR), and balance. Middle-aged women can decrease their WHR by engaging in regular, long-term Tai Chi Chuan practice since it is a type of whole-body exercise that can enhance shape, lower body fat percentage, increase lean body mass, and ultimately lower WHR (Ningbo and Long, 2008). Tai Chi Chuan practitioners learn to maintain balance in their bodies through slow, flowing motions, which not only develops the core muscles but also improves body coordination. This balance practice is very essential in preventing falls and injuries among the elderly (Bai et al., 2024). Accordingly, Tai Chi Chuan has an obvious the advantage over other workouts in increasing flexibility and balance.

Jiamusi gymnastics had a statistically influence in RHR, when compared with other exercise groups. Adults typically have a resting heart rate between 60 and 100 beats per minute. Lower resting heart rates are generally associated with improved cardiovascular health and more effective cardiac function (Olshansky et al., 2023). Jiamusi gymnastics, a type of aerobic exercise appropriate for middle-aged and senior persons, raises the heart rate and respiratory rate to enhance oxygen intake through precise movements and rhythms, promotes blood circulation, and aids in the prevention of cardiovascular illnesses. Research have showed that middle-aged and older ladies can increase their cardiovascular system’s performance and their lungs’ ventilation function by practicing square dancing regularly for 6 months (Jiang, 2022). Thus, Jiamusi gymnastics has a greater impact on cardiovascular system’s performance and their lungs’ ventilation function than Tai Chi Chuan and walking. In addition, Jiamusi gymnastics can be the first option for long-term exercise for those with cardiovascular disease.

Walking had a statistically effect on chair stand test (CS) compared to other exercise groups. Walking causes the gastrocnemius muscle to tighten, which propels the body forward. The calf gastrocnemius muscle can be strengthened with repeated use of this contraction. Second, rapid walking strengthens the entire lower limb system, enhances joint flexibility, tones the calf muscles, and successfully staves against leg senility (Yuxiu, 2023). It is not unexpected that leg strength is associated with longevity because it frequently reflects one’s level of activity. Individuals who engage in greater physical activity have a lower risk of obesity and major chronic illnesses like diabetes, hypertension, and high cholesterol (Booth et al., 2012). Research had revealed that seniors with moderate to high levels of walking can strengthen their legs. Consequently, middle-aged and older people’s overall health depends on their leg strength, which can be developed by regularly engaging in moderate-to-intense walking.

## Limitation

It is common knowledge that individuals who exercise regularly have varying diets and regimens, as a result, discrepancies in the results may occur. However, HGS and CS are trustworthy evaluation indicators in the majority of the studies in this study. It would be beneficial for future researchers to expand on the findings of this study by more precisely evaluating muscle endurance testers and monitoring physiological indicators. In addition, all the study’s participants were female and ranged in age from 50 to 70. Future research should focus on determining whether this finding applies to men in this age range. Furthermore, under typical conditions, self-reported data such exercise duration, frequency, and cycle have a low degree of reliability. However, the person in charge of the exercise project was consulted prior to the start of the study survey data, and the team members’ information and the exercise conditions were in agreement with what the exercisers said.

## Conclusion

To summarize, the research indicates that Tai Chi Chuan, walking, and Jiamusi gymnastics can be utilized as appropriate forms of exercise to enhance the health risks associated with middle-aged and older women while also improving their physical and emotional well-being. In addition, the majority of groups choose these three forms of exercise because they are easy to do, safe, and do not require any specific equipment. This is significant because it will support China and other nations in achieving healthy aging. We believe that the scientific community has sufficient evidence to support the benefits of these three exercise approaches; however, in order for middle-aged and older participants to comprehend the fundamental elements of each movement to further enhance its functionality, more professional athletes are required to mentor and encourage them.

## Data Availability

The original contributions presented in the study are included in the article/supplementary material, further inquiries can be directed to the corresponding authors.
